# 

**Published:** 1864-03

**Authors:** 


Volume XXI. Number 3.

### THE CHICAGO

### MEDICAL JOURNAL.

DE LASKIE MILLER, M. D.,

EPHRAIM INGALS, M. D.,

Editors and Proprietors.

MARCH, 1864.

CHICAGO:

J. C. W. BAILEY, BOOK AND JOB PRINTER, 130 CLARK STREET.

1864.

